# Characterization and phylogenetic analysis of multidrug-resistant protein - encoding genes in *Trypanosoma evansi* isolated from buffaloes in Ngawi district, Indonesia

**DOI:** 10.14202/vetworld.2019.1573-1577

**Published:** 2019-10-17

**Authors:** Mohammad Mirza Nuryady, Rini Widayanti, Raden Wisnu Nurcahyo, Brilyantika Fadjrinatha, Ahmad Fahrurrozi Z. S

**Affiliations:** 1 Department of Biology Education, Faculty of Teacher Training and Education, Universitas Muhammadiyah Malang, Malang, Indonesia; 2 Department of Biochemistry, Veterinary Medicine Faculty, Universitas Gadjah Mada, Yogyakarta, Indonesia; 3 Department of Parasitology, Veterinary Medicine Faculty, Universitas Gadjah Mada, Yogyakarta, Indonesia; 4 Department of Tropical Medicine, Medicine Faculty, Universitas Gadjah Mada, Yogyakarta, Indonesia

**Keywords:** multidrug-resistant protein gene, phylogenetic analysis, surra, *Trypanosoma evansi*

## Abstract

**Background and Aim:**

Excessive use of trypanocidal drugs can lead to cases of drug resistance. Multiple cases of resistance have been widely reported for drugs such as isometamidium chloride and diminazene aceturate. These cases deserve serious attention, especially in Indonesia, where the first case was recorded and where the molecular basis of trypanocidal drug resistance has never been evaluated. This study aimed to analyze the multidrug resistance protein (MRP) gene in *Trypanosoma evansi* isolates, sampled from Indonesia, by focusing on the phylogenetic relationship between these isolates and other *Trypanosoma* spp.

**Materials and Methods:**

A total of 88 blood samples were drawn from buffaloes in the Ngawi district, Indonesia. Animals infected with *T. evansi* were detected through the microhematocrit technique and Giemsa blood smear methods. Positive blood samples were used to inoculate in male mice (*Mus musculus* BALB-C strain) as an animal model for culturing the *T. evansi*. The genomic DNA of the blood taken from the *T. evansi-* infected mice was used for polymerase chain reaction amplification, sequencing, and phylogenetic analysis.

**Results:**

Two genes were analyzed; the first gene detected for *T. evansi* corresponded to *Trypanosoma* *brucei* with a homology of 99% and the second gene to *Trypanosoma brucei gambiense*, with a homology of 100%. These two genes of the MRP from *T. evansi* showed clear similarity to the *MRPE* and *MRPA* genes of the *T. brucei* ssp.

**Conclusion:**

The *MRP* gene is conserved on the subspecies level of *T. brucei*. Only few point mutations were found between various sequences, which mean that the proteins have the same structure. This is important to treat the parasite with the appropriate drugs in the future.

## Introduction

*Trypanosoma evansi* has the widest host range among all the pathogenic trypanosomes and infects both domestic animals and wildlife throughout the world. *T. evansi* is the cause of the infectious multispecies disease called surra [1]. The importance of controlling surra has been identified by the Indonesian government, and they have subsequently established surra as a strategic infectious animal disease in 2012. The prevention and the eradication of the disease are generally focused on two areas: Pathogen control and vector control [2]. The government implemented a treatment program using trypanocidal drugs such as isometamidium (Trypamedium^®^, Samorin^®^) and diminazene (Bereni^l®^, Tryponi^l®^) in an effort to control the disease [3].

It is important to note that the excessive use of trypanocidal drugs can lead to resistance among *T. evansi* isolates. The resistance to trypanocidal drugs has been widely reported in several countries [3,4]. There are three commonly used trypanocidal drugs that can lead to drug resistance: Isometamidium chloride (ISM), diminazene aceturate, and homidium. There are an escalating number of cases showing an increase in resistance against these trypanocidal agents. Research by Tihon *et al*. [5] showed that continuous treatment with ISM will lead to changes in the adenosine triphosphate-binding cassette (ABC) transporter coding gene as well as other membrane transporter proteins. The ABC transporters are one of the largest protein families present in organisms and have been found to play a critical role in multidrug resistance [6,7]. The multidrug resistance protein (MRP), a subfamily of the ABC transporters, might potentially be influenced through continuous exposure to ISM [8,9]. However, at this stage, no member of this ubiquitous protein family has yet been described in *T. evansi*.

Cases of surra in Indonesia deserve particular attention since the first recorded case was in Indonesia. In addition, it would also be beneficial to further study cases of multidrug resistance in *Trypanosoma* to contribute to the understanding of the roles of MRP in *T. evansi*. This study aimed to analyze the *MRP* gene in *T. evansi* isolates, sampled from Indonesia, by focusing on the phylogenetic relationship between these isolates and other *Trypanosoma* spp.

## Materials and Methods

### Ethical approval

All experimental protocols and animal work were approved by the Ethical Clearance Committee of the Veterinary Faculty, Gadjah Mada University, Indonesia (clearance number 0024/EC-FKH/Int./2018), and conducted with strict adherence to the principles of laboratory animal care.

### Trypanosome sample collection

In total, 88 blood samples (56 females and 32 males) were drawn from buffaloes in the Ngawi district, Indonesia, and brought to Balai Besar Veteriner Wates. All 88 blood samples were analyzed using microhematocrit and Giemsa staining of blood smears to determine the presence of *T. evansi* in the blood samples. The microhematocrit centrifugation technique method was done by taking blood from mice infected with microhematocrit capillaries and centrifuging it at 2555× *g* for 10 min; then, the *Trypanosoma* were observed in the white area, which is called the buffy coat. One positive sample was randomly selected and processed as described in section 2.4; 5 ml of the diluted solution was subsequently injected into the peritoneal cavity of 20 male mice (*Mus musculus* BALB-C strain), which were used as the animal model for the study.

### Maintaining animal models

The 20 adult male mice had a weight range of 25-30 g. These mice were obtained from the Animal House Research Center of Gadjah Mada, Indonesia. The mice were allowed to acclimatize to their environment for 14 days in the parasitological laboratory of the veterinary faculty of the university, in which the experiment was conducted. The mice were housed in plastic cages under standard hygienic conditions with wood shavings as bedding which was changed weekly. The mice were kept at the ambient temperature of 24-26°C and relative humidity of 70-80%, with a 12 h/day light period. They were provided with rat pellets and water *ad libitum*.

### Parasitic inoculation of mice

An infected blood sample (1 ml) was drawn from a donor mouse at peak parasitemia and diluted in 4 ml physiological saline solution, and these dilutions were also used to maintain the samples. Subsequently, 5 ml of the diluted blood sample was injected into the peritoneal cavity of healthy mice. One milliliter of the blood containing approximately 10^6^ cells/ml was inoculated into each mouse at 30 mg body weight [5]. The parasitemia was monitored daily by preparing a wet mount which was then viewed under a light microscope. Mice in which a parasitemia of 10^5^ cell/blood slide was observed were identified, and blood was drawn through the retro-orbital sinus after which they were euthanized. Blood samples were stored at 4°C to be used for DNA extraction.

### DNA extraction

DNA was extracted from the mouse blood samples containing *Trypanosoma* using the DNeasy DNA extraction kit (Qiagen) according to the manufacturer’s instructions. Phosphate-buffered saline was added to 150 ml of the blood sample until a final volume of 200 µl and mixed gently. Twenty microliter proteinase K was added to the solution, vortexed, and incubated at 60°C for 5 min, during which time, the tube containing the solution was inverted every 2 min. To the solution, 200 µl absolute alcohol was added, and the sample was vortexed for 10 s. The total volume of the solution was transferred to a spin column and centrifuged at 14-16,000× *g* for 1 min. Following centrifugation, an internal membrane was moved to the new collection column. To the DNA-binding column, 400 µl of W1 buffer was added followed by centrifugation at 14-16,000× *g* for 30 s. The DNA-binding column was washed by the addition of 600 µl wash buffer and centrifuged at 14-16,000× *g* for 3 min. The DNA was eluted by the addition of 100 µl buffer elution and recovered by centrifugation at 14-16,000× *g* for 1 min.

### DNA amplification

Each polymerase chain reaction mixture was performed in a final volume of 50 µl and contained 25 µl of 2× GoTaq Green polymerase Master Mix, 10 µl DNA template extracted as described in section 2.5, 1 µl genome-specific forward and reverse primers, and distilled water to the final volume. Subsequently, the tubes were placed into a thermal cycler and subjected to an initial denaturation at 94°C for 7 min, followed by 35 cycles of denaturation at 94°C for 30 s, primer annealing at 58°C for 30 s, and extension at 72°C for 60 s. Following the last cycle, the reaction was kept at 72°C for 5 min to allow time for the completion of all the strands [10]. The primers used for the reaction were specific for the *MRP* gene of *Trypanosoma brucei* F1 5’ CCTCACTTTGTGGCCGTATAA 3’, primer R1 5’ ATCCACAGCCTCCGTATTCTT 3’; primer F2 5’ AGATGAGTGATCAAGACTGGAAA 3’, primer R2 5’ ACCGGTTATACCACCATTA CTTT 3’.

### DNA sequencing and phylogenetic analysis

The amplified DNA sequences were submitted for sequencing at the sequencing facility in First Base, Malaysia, and sequenced using the forward and reverse primers (section 2.6). The nucleotide sequences were analyzed and compared with the *MRP* gene sequence of *T. brucei* as a positive control (GenBank accession number XM841956.1). Phylogenetic trees were constructed using maximum likelihood with the 2-parameter Kimura model of MEGA 7 software [11,12].

## Results

### Morphology

**Figure-1:**
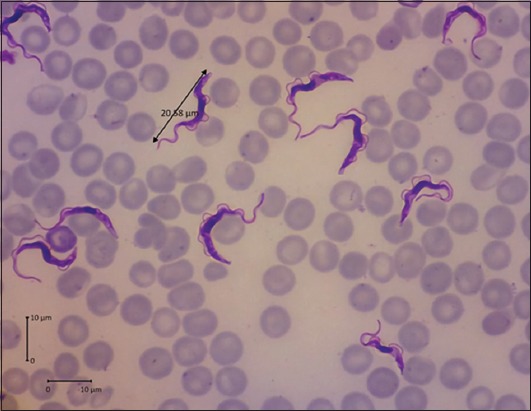
Morphology of *Trypanosoma evansi* with Giemsa staining viewed under a light microscope (original 400×).

The morphology of the *Trypanosoma* isolates present in the buffaloes blood samples from Ngawi (East Java Province) matched that of *T. evansi* (Figure-1) as described by Desquesnes *et al*. [1].

### DNA sequence analysis, variation, and nucleotide diversity

Results for the first *T. evansi MRP* gene showed 99% similarity of query coverage to the *T. brucei* *MRPA* (*TbMRPA*, Tb927.8.2160). It was approved as an *MRPA* gene of *T. evansi* and named the *T. evansi* *MRPA* gene (*TevMRPA*). A subsequent analysis of the variation between *TevMRPA* and *TbMRPA* revealed the presence of several mutations in *TevMRPA* (Table-1). Two categories of mutations were present; three instances of transition mutations and nine instances of transverse mutations. However, the nucleotide diversity between *TevMRPA* and *TbMRPA* was 1.3% indicating that the nucleotide variation was not significant.

**Table 1:** Comparison of nucleotide variations and types of mutations between *TevMRPA* and *TbMRPA*.

Species	Nucleotide sequence number											

	51	72	153	193	311	428	456	583	792	777	860	903
*TevMRPA* gene	A	G	G	A	G	G	A	T	T	T	A	A
*TbMRPA* gene	G	A	A	C	A	A	G	A	C	G	G	G
Type of mutation	TS	TS	TS	TV	TS	TS	TS	TV	TS	TV	TS	TS

*Description: TV and TS. TV=Transverse mutation, TS=Transition mutation, *TevMRPA*=*Trypanosoma evansi* *MRPA* gene, *TbMRPA*=*Trypanosoma brucei* *MRPA*

The second *T. evansi MRP* gene that was sequenced showed 100% similarity to the *Trypanosoma brucei gambiense* *MRPE* gene (*TgMRPE*, XM 011774747.1). Confirming that, this gene was a *MRPE* gene from *T. evansi*, and it was named the *T. evansi* *MRPE* gene (*TevMRPE*). The subsequent analysis of the variation between *TevMRPE* and *TgMRPE* revealed only one transition mutation at 766 bp where adenosine was substituted for guanine in the *TevMRPE* nucleotide sequence. Further analysis showed that this mutation did not lead to any change in amino acid sequence of *TevMRPE*. The nucleotide diversity between *TevMRPE* and *TgMRPE* was 0.07%, which was not significant.

### Inter- and intra-species diversity with phylogenetic analysis

**Figure-2:**
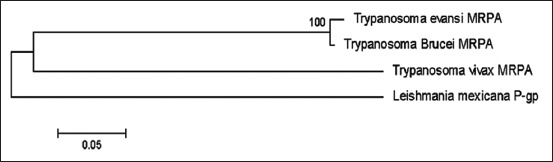
Phylogenetic tree of the *Trypanosoma evansi* MRPA gene (*Trypanosoma evansi* MRPA) gene using maximum likelihood with the 2-parameter Kimura model. The phylogenetic tree showed that *TevMRPA* and *Trypanosoma brucei MRPA* are in one clade.

**Figure-3:**
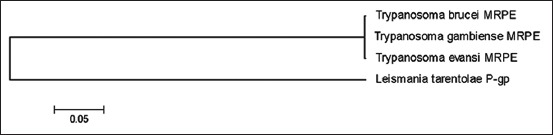
Figure-3: The *Trypanosoma evansi MRPE* gene (*TevMRPE*) gene phylogenetic tree uses the construct maximum likelihood tree with the 2-parameter Kimura model. The phylogenetic tree showed that *Trypanosoma MRPE*, *Trypanosoma gambiense MRPE*, and *Trypanosoma brucei MRPE* are in one clade.

The *Tev* MRP partial genes were compared to several intraspecies and interspecies the MRP family genes such as *MRPA*, *MRPE*, and *P-gp* gene using discontinuous megablast results. The phylogenetic tree (Figure-2) showed that the *TevMRPA* gene had a short genetic distance to *TbMRPA* (1.4%) and a large genetic distance to *Trypanosoma vivax* *MRPA* (*TvxMRPA*) and *Leishmania mexicana* *P-gp* (*LmPgp*), 47% and 50%, respectively (Table-2). The maximum likelihood tree of *TevMRPA*, constructed with the Kimura 2-parameter method showed that *TevMRPA* was included in a clade along with *TbMRPA*, while the *TvxMRPA* and the *LmPgp* were included in different clades (Figure-2). The phylogenetic tree for *TevMRPE* (Figure-3) showed that the branch between *TevMRPE*, *TgMRPE* (0.1%), and *TbMRPE* (0.3%) was short, and there was a large genetic distance of 72% between *TevMRPE* and *Leishmania tarentolae P-gp* (*LtPgp*) (Table-3). In addition, it was found that *TevMRPE*, *TbMRPE*, and *TgMRPE* were in one clade, whereas *LtPgp* were altogether in a different clade (Figure-3).

**Table 2:** Genetic distance of the *Trypanosoma evansi MRPA* gene compared with intraspecies and interspecies.

Species	1	2	3
*Trypanosoma evansi* *MRPA*			
*Trypanosoma brucei* *MRPA*	0.014		
*Trypanosoma vivax* *MRPA*	0.470	0.467	
*Leishmania mexicana* *P-gp*	0.504	0.490	0.542

**Table 3:** Genetic distance of the *Trypanosoma evansi MRPE* gene compared with intraspecies and interspecies.

Species	1	2	3
*Trypanosoma evansi* *MRPE*			
*Trypanosoma brucei MRPE*	0.003		
*Trypanosoma brucei gambiense* *MRPE*	0.001	0.001	
*Leishmania tarentolae* *P-gp*	0.725	0.725	0.721

## Discussion

There have been several cases of *T. evansi* drug resistance reported in Indonesia [13]. Such cases of resistance to trypanocidal drugs can be a result of mutations in the gene responsible for the transporter proteins in cell membranes and organelles [14,15]. This was investigated by Campos *et al*. [16], in which they exposed *Trypanosoma cruzi* to Benznidazole. In their study, they found that continuous exposure of *T. cruzi* to Benznidazole resulted in mutations in a novel ABCG-like transporter coding gene, which caused an almost 2-2.6-fold increase in the resistance of *T. cruzi* to Benznidazole [16,17]. Drug resistance that results from mutations may lead to the transporter proteins selectively transporting substances across the cell membrane preventing the trypanocidal drugs from entering the cell. Based on the role of MRP in *Trypanosoma* spp., it was of interest to specifically investigate MRP in *T. evansi* [5,9].

Research by Shahi *et al*. [18] showed that overexpression of the *MRPE* gene would cause a 2-3-fold increase in suramin drug resistance, followed by an increase in the efflux work by *MRPE*. Suramin is a medication used to treat African Trypanosoma. Suramin is a highly charged molecule at physiological pH and, therefore, unable to permeate the cell membrane by passive diffusion. It is administered by intravenous injection. The mechanism of action for suramin remains unclear; however, it is thought that parasites are able to selectively uptake suramin through receptor-mediated endocytosis of drugs bound to low-density lipoproteins and, to a lesser extent, other serum proteins. Once inside parasites, suramin combines with proteins, especially trypanosomal glycolytic enzymes, to inhibit energy metabolism [19]. It has been reported that overexpression of *T. cruzi* *MRPA*, also known as the ABCC transporter, was related to chemotherapy failure [20].

Nucleotide changes in the *TevMRPA* gene as compared to the *TbMRPA* gene impact the translation of the sequences to amino acids; there were six amino acid changes observed between these two genes. Such a change could possibly alter the protein structure and function; however, there are certain instances where such a mutation does not have an impact on the amino acid sequence, for example, cases where isoleucine is changed to leucine. An example of amino acid alterations that could give rise to differences in the function of the protein is changed in arginine. Arginine, which is a component of charged proteins (located in the side chains of salt bridges) changed to histidine would lead the different function of the protein, which is usually histidine, binds the hydrogen and functions as proton donor or acceptor [21]. Another example of such a case is the change from isoleucine to methionine, which would change the structure of the protein itself [22]. Research by Mathieu *et al*. [23] showed that the increasing number of arginine and lysine amino acids in the transporter proteins is essential for *T. brucei*. On the other hand, the *TevMRPE* gene has only one nucleotide difference when compared to *TgMRPE*. In addition, there is no change in amino acids, indicating that the *MRPE* gene is conserved at the subspecies level in *T. brucei*.

The results of the genetic distance and phylogeny analysis showed that the putative *Tev* MRP gene is always in one clade with the *MRP* gene from *T. brucei* spp. Moreover, *T. evansi* and *Trypanosoma gambiense* most likely evolved from *T. brucei* spp.; hence, *T. evansi* is also called *T. brucei evansi*. There are several subspecies in *T. brucei*, such as *T. brucei*, *T. brucei gambiense*, *T. brucei evansi*, and *T. brucei equiperdum* [1]. Wen *et al*. [24] reported that *T. evansi* and *T. equiperdum* are subspecies of *T. brucei*. The evolution is based on the mitochondrial/kDNA, which is the distinguishing factor between the various *T. brucei*. The *T. brucei* has hundreds of minicircles, while *T. equiperdum* and *T. evansi* only have one minicircle [25].

## Conclusion

The obtained genes were putative as *TevMRPA* and *TevMRPE*. In this case, the coding gene of the MRP was conserved on the subspecies level; only a few point mutations were found between various sequences, which mean that the proteins have the same structure. Future research can be conducted on further understanding of the drug resistance mechanism.

## Authors’ Contributions

MMN and RW performed the experiments and analyzed the data. BF and AFZS helped in performing the experiments. MMN, RW, and RWN designed the study and coordinated the work and wrote the manuscript. All authors read and approved the final manuscript.
